# 

**Published:** 1853-09

**Authors:** 


### CONTENTS

### OF NO III.

### ORIGINAL COMMUNICATIONS.

### ESSAYS AND CASES.

RT. I.—A. Case of Ovariotomy. By J. TAYLOR BRADFORD, M. D., of Augusta, Kentucky. - - 185

ART. II.—A Case of Displacement of the Heart, in consequence of Chronic Disease of the Chest. By W. C. SNEED, M. D., Secretary Kentucky State Medical Society, Frankfort, Kentucky. - - - 188

ART. III.—A Case of Gangrene, following laceration of the Popliteal Artery and Nerve. By M. SHEPHERD, M. D., of Payson, Ills. - - - - - 193

### REVIEWS.

ART. IV.—A Practical Treatise on Diseases of the skin. By J. MOORE NELIGAN, M. D., M. R. I. A., Honorary Fellow of the Society of Physicians of London, Physician to the Jarvis Street Hospital, and Lecturer on Practice of Medicine in the Dublin School of Medicine. - 196

ART. V.—The Claims of the Medical Profession. The Annual address Delivered before the New York State Medical Society and Members of the Legislature, at the Capitol, February, 1853, by A. CLARK, M. D., President of the Society, and Professor of Physiology and Pathology in the College of Physicians and Surgeons of the State of New York, etc. - - - - - - 215

SELECTIONS FROM FOREIGN AND HOME JOURNALS.

Some Account of a Fever produced by the Decomposition of Potatoes, in the Village of Almont, Michigan. - - 221
Consumption and Malaria. - - - - - 227
Ovaritis. - - - - - - - 228
Disease of Women Unconnected with Pregnancy. - - 228
Phlegmasia Dolens Relieved by Cold Water. - - 229
On Anesthetic Inhalations in Certain Painful Affections of the Chest. - - - - - - - - 231
Statistics of Operations for Cancer. . - - 232
On the Application of Nitrate of Silver in Acute Tonsillitis. - 233
Air-Compressor of the Testicle. - - - - 234
MM. Medicus and Physiologus on the Development of Intestinal Worms. - - - - - - - 236
Ether and Chloroform. - - - - 240
On the Application of Gutta Percha in the Treatment of Diseases of the Skin. - - - - 242
Galactagogue and Emmenagogue Effects of warm and stimulating Applications to the Mammæ. - - - 249
Further Researches on the Pathology of phlegmasia Dolens. 256
Medical Properties of Ox Gall. - - - 263
On the Treatment of Ascites by Intra-peritoneal Injections. - 266

ORIGINAL INTELLIGENCE.

The Weather and Diseases. - - - - - - 269
Professor Siliman. - - - - - - - - 270
New Medical Journals. - - - - - - - 271
Epidemics—Circular. - - - - - - 272
Ovariotomy. - - - - - - - - - 272
A New Work on Anatomy. - - - - - - - 273
Essays and Addresses. - - - - - - - 273
Cholera. - - - - - - - - - 274
Receptionof Dr. Marshall Hall by the Buffalo Medical Association. 274
Prize Essays. - - - - - - - - - 276

